# 

**Published:** 1916-06

**Authors:** 


### OUR NEW SUBSCRIBERS

Dr. A. M. Moore------W. Va.
Dr. C. L. Murphree-------Ala.
Dr. H. H. Glover----------Ga.
Dr. F. D. Bradford------Ala.
Dr. H. F. Harris---------Ala.
Dr. J. H. Blackwell------Ala.
Dr. S. M. Withers---------Ga.
Dr. E. R. Gnassi---------Ala.
Dr. Thos. Collins--------Ala.
Dr. H. Buck---------------La.
Dr. J. C. Davis----------Fla.
Dr. R. F. Goddard--------Fla.
Dr. A. B. McKinzie------Ala.
Dr. G. A. Weaver---------Ala.
Dr. A. O. Campbell------Fla.
Dr. J. R. Folmar---------Ga.
Dr. L. A. Cockfield---------La.
Dr. C. P. Rutledge---------La.
Dr. M. D. Thomas---------Ala.
Dr. W. W. Smith---------La.
Dr. W. P. Allen----------Ga.
Dr. Paul H. Ringer------N. C.
Dr. W. L. Grimes------N. C.
Dr. Jas. W. Hooper----N. C.
Dr. W. L. Grantham----N. C.
Dr. W. H. Adams---------Fla.
Dr. Addison Brenizer----N. C.
Dr. H. M. Campbell----W. Va.
Dr. Robt. T. Felts------N. C.
Dr. J. G. Raby----------N. C.
Dr. O. K. Phligar---------Va.
Dr. E. P. Merritt----------Ga.
Dr. J. L. Hassell------N. C.
Dr. A. H. Herr---------Okla.
Dr. W. J. Holton---------Fla.
Dr. C. W. Maxwell------S. C.
Dr. D. S. Edwards------Texas
Dr. G. L. Johnson---------Fla.
Dr. C. E. Grimm------W. Va.
Dr. R. A. Ely--------------Fla.
Dr. F. S. Richmond----W. Va.
Dr. A. L. Bryan----------Fla.
Dr. J. W. Allen---------S. C.
Dr. Geo. Fordham----W. Va.
Dr. E. H. Stahl------W. Va.
Dr. S. L. Booker------N. C.
Dr. J. D. Jacobs----------Ga.
Dr. S. D. McPherson----N. C.
Dr. Chas. A. Borey------La.
Dr. Bates McClure----W. Va.
Dr. J. D. Bearden------S. C.
Dr. E. B. Glenn---------N. C.
Dr. O. F. Gober---------Texas
Dr. Earl Ferguson------Texas
Dr. J. C. Carter---------W. Va.
Dr. D. C. Northcross----Ala.
Dr. D. G. Preston----W. Va.
Dr. R. E. Houke------W. Va.
Dr. S. P. Combs-----------Ky.
Dr. A. B. Jemison---------Ga.
Dr. R. L. Fauy---------W. Va.
Dr. L. A. Williams----W. Va.
Drs. Jones & Bickley---Texas
Dr. W. A. Gault---------Texas

Dr. L. D. Conn--------Texas
Dr. E. W. Crook------W. Va.
Dr. J. M. Grantham------Fla.
Dr. F. L. Erwin------W. Va.
Dr Thos. M. Geen------N. C.
Dr. S. A. Morris---------Fla.
Dr. Wm. R. Warren------Fla.
Dr. J. Ross Hunter----W. Va.
Dr. H. M. Batson------W. Va.
Dr. N. A. Upchurch------Fla.
Dr. W. K. Walker------S. C.
Drs. Stilley & Washburn--Ky.
Dr. R. A. Barnett--------Fla.
Dr. G. C. Balke------W. Va.
Drs. Bell & Smith------Texas
Dr. L. L. Andrews------Fla.
Dr. Chas. R. Wooline--W. Va.
Dr. C. E. Reitzel--------N. C.
Dr. Ira M. Fisher------W. Va.
Dr. M. W. Skaggs------Fla.
Dr. J. M. Bannister----Texas
Dr. E. R. Carpenter----Texas
Dr. H. S. Swope----------Ky.
Dr. David White-------Texas
Dr. L. M. Cleckley--------Ga.
Dr. J. H. Jerigan-------Texas
Dr. H. E. Steverson----Texas
Dr. Rollin Jefferson-------Fla.
Dr. J. R. Fridge----------La.
Dr. L. B. Mitchell--------Fla.
Dr. C. P. Shirkey------W. Va.
Dr. J. D. S. Davis--------Ala.
Dr. D. S. Porter--------S. C.
Dr. J. W. Shoemaker----Texas
Dr. E. H. Walet----------La.
Dr. Nathan Boyd-----N. Mex.
Dr. A. J. Pickering--W. Va.
Dr. Jno. Q. Myers------N. C.
Dr. I. H. Archer--------N. C.
Dr. W. D. Roussel--------La.
Dr. Harold Glasscock----N. C.
Dr. C. W. Lemon------W. Va.
Dr. J. H. Cannon------S. C.
Dr. D. Abshire--------W. Va.
Dr. H. N. Mayes---------Miss.
Dr. J. D. Manget---------Ga.
Dr. T. N. Reid----------N. C.
Dr. J. C. Herrington----Miss.
Dr. H. L. Hill----------Okla.
Dr. Jno. S. Clifford------N. C.
Dr. C. B. Preston-----W. Va.
Messrs. E. W. Allen & Co.--Ga.
E. B. Mitchell------------Ga.
L. B. Daly---------------Ga.
Alfred Brown------------Ga.
R. C. Turck--------------Fla.
Thos. H. Coble---------Texas
G. C. Parker--------------Va.
S. P. Walker----------W. Va.
R. F. Harrell-------------La.
D. D. Stauss------------S. C.
F. L. Carpenter---------S. C.
A. M. Jackson-----------Fla.
S. E. Johnson-----------Fla.
J. F. Corry------------Texas
A. T. Neely------------S. C.
Harrison & Park----------Ga.
J. H. Thomas-----------S. C.
LeRoy Willis--------------Ky.
Alice E. Johnson-------N. C.
J. G. Mock-------------S. C.
W. H. Wallingford----W. Va.
A. G. Holmes------------Fla.
P. H. Jones---------------La.
W. L. Stringer-----------Ala.
Sarah T. Mayo------------La.
V. M. Long-------------N. C.
T. F. Garrett----------W. Va.
Kessler & Gorke------W. Va.
F. C. Shute---------------La.
J. K. Johnston-----------Fla.
F. G. DuBose------------Ala.
A. L. Morrison--------W. Va.
Simmons & Stafford-------La.
P. K. Switzer-----------S. C.
City. Hos. for Ment. D.--La.
T. E. White--------------Ga.
R. A. Quinn------------Miss.
C. E. Cotton------------N. C.
A. L. Atwood------------Ala.
Earl McCracking----------La.
W. R. Brandon----------N. C.
J. M. Mason-------------Ala.
O. J. Whitcomb------N. Mex.
J. L. Duckett----------S. C.
Dr. Raymond Pollock--N. C.
T. J. Fleming-------------La.
J. C. Moore------------N. C.
T. F. Oates------------Texas
R. W. Timberlake------W. Va.
B. T. Wise---------------Ga.
Dr. A. O. Williams--------Ky.
Dr. N. S. Lea-----------S. C.
Dr. Chason & Chason------Ga.
Dr. O. P. Nuckols--------Ky.
Dr. J. K. Cowherd----W. Va.
Dr. Wm. P. Adamson------Fla.
Dr. E. F. Hayden------Okla.
Dr. R. H. McGinnis------Fla.
Dr. R. H. Dean-----------Fla.

